# A Simple Matlab Code for Material Design Optimization Using Reduced Order Models

**DOI:** 10.3390/ma15144972

**Published:** 2022-07-17

**Authors:** George Kazakis, Nikos D. Lagaros

**Affiliations:** Institute of Structural Analysis and Antiseismic Research, School of Civil Engineering, National Technical University of Athens, 9, Heroon Polytechniou Str., Zografou Campus, GR-15780 Athens, Greece; kzkgeorge@gmail.com

**Keywords:** topology optimization, microstructure, homogenization, Matlab, reduced order models, reduced basis, on-the-fly construction, POD, approximate reanalysis

## Abstract

The main part of the computational cost required for solving the problem of optimal material design with extreme properties using a topology optimization formulation is devoted to solving the equilibrium system of equations derived through the implementation of the finite element method (FEM). To reduce this computational cost, among other methodologies, various model order reduction (MOR) approaches can be utilized. In this work, a simple Matlab code for solving the topology optimization for the design of materials combined with three different model order reduction approaches is presented. The three MOR approaches presented in the code implementation are the proper orthogonal decomposition (POD), the on-the-fly reduced order model construction and the approximate reanalysis (AR) following the combined approximations approach. The complete code, containing all participating functions (including the changes made to the original ones), is provided.

## 1. Introduction

The basic theory for the implementation of topology optimization in material design was presented first in 1994 by Sigmund [1], followed by Sigmund and Torquato in 1997 [2] and by Gigiansky and Sigmund in 2000 [3]. Since then, many other studies have been published dealing with a variety of different material optimization problem formulations. Neves et al. [4] and Fujii et al. [5] used the density-based approach to design periodic microstructures for optimal elastic properties. Guest and Prévost [6] dealt with the topology optimization of the fluid flows in the design of porous periodic materials. Challis et al. [7], Amstutz et al. [8] and Gao et al. [9] proposed level set-based approaches for the design of microstructures, and Huang et al. in [10,11] presented a Bi-directional Evolutionary Structural Optimization (BESO)-method-based approach for the optimal design of periodic microstructures. A detailed review of the different methodologies in the optimal design of materials together with a description of the variety of the approaches presented so far to deal with the topology optimization of the macro design concurrently with the micro design can be seen in [12].

In the past, due to the increased computational effort required for solving the topology optimization problem, various methodologies along different directions (approximate reanalysis, model order reductions, machine learning, etc.) have been presented. Indicatively, Kirsch and Paralambros [13] first proposed a unified approach to structural reanalysis using the combined approximations in topology optimization. Wang et al. [14] presented a methodology of recycling search spaces in iterative solvers during the optimization procedure. Amir et al. [15] proposed an approximate reanalysis approach in topology optimization based on the combined approximations approach and the use of approximations for dealing with the solution of the analysis problem, generated by a Krylov subspace iterative solver [16]. In addition, in [17], Amir et al., addressed the computational cost of the robust topology optimization formulation. Gogu [18] presented an on-the-fly approach for the construction of the reduced order model. Alaimo in [19] proposed an reduced order model approach where a reduced basis is created based on the functional principal component analysis (FPCA). Ferro et al. [20] proposed a proper orthogonal decomposition (POD) approach where the stages of the SIMP method were used as reduced basis vectors during the optimization procedure. Senne et al. [21] proposed a combination of the approximate reanalysis technique with the sequential piecewise linear programming method, and Xiao et al. [22] proposed a reduced order modeling approach which constructed the reduced basis using the proper orthogonal decomposition (POD) approach. Meanwhile, in the same direction, to reduce the computational effort, various machine learning methodologies have been presented, and the precursor of these was a study by the authors [23].

So far, many Matlab code implementations of the topology optimization formulation have been presented in various publications. For the density-based approach, the first code was the so-called top99 [24] implementation that was followed by the top88 one [25]. Both Matlab codes were dealing with the 2D topology optimization problem formulation. Liu et al. [26] and Ferrari [27] presented an extension of the density-based approach into the 3D space, with Ferrari [27] suggesting code modifications for achieving better performance. Talischi et al. [28] and Chi et al. [29] expanded the 2D and 3D density-based approaches by using the capability to deal with unstructured meshes as well. Amir et al. in [30] presented a code implementation for improving the computational cost of the topology optimization procedure using the multi-grid, preconditioned conjugated gradients solver (MGCG). Huang et al. [31] presented an Evolutionary Structural Optimization (ESO) topology optimization code implementation based on the top99 for the 2D space. Wang et al. [32] and Challis [33] published code implementations that rely on the level set approach for the topology optimization for 2D problem formulations. Otomori et al. [34] and Wei et al. [35] also presented level set-based code implementations for the topology optimization using the reaction diffusion equation and radial basis functions, respectively. In 2019, an integration of a topology-optimization procedure with SAP2000, well-known commercial software for analysis and design of structural systems, was presented by the authors [36]. In addition, Gao et al. in [37] presented IgaTop, a topology optimization formulation using isogeometric analysis.

Subsequently, several code implementations were also presented that were dealing with the homogenization-based topology optimization approaches. Specifically, numerical homogenization implemented for 2D and 3D material design was presented in studies [38,39]. In addition, an energy-based homogenization approach combined with the optimal design of materials was presented in [40]. In this study, a topology-optimization-based Matlab code implementation is presented that deals with the problem of material design at the microstructure level, assisted by model order reduction (MOR) approaches. In particular, the topology optimization procedure is combined with the proper orthogonal decomposition (POD), the on-the-fly reduced order model construction and the approximate reanalysis following the combined approximations approach. Although the code provided covers the case of microscale material design for 2D design domains, it can easily be extended to 3D design domains by modifying the homogenization part to produce the 3D elasticity tensor of the unit cell and extend the problem formulation’s description to handle both macro and micro scales. The implementation of the MOR approaches is independent of the dimensionality of the formulation due to being applied in the solution part of the finite element analysis performed at the macro scale.

The layout of the work is composed of four sections accompanied by Section 1 and Section 6. In particular, a short description of the optimal design problem of materials is presented in Section 2; subsequently, in Section 3 the theoretical part of the integration of the model order reduction methodologies into the material optimization problem is provided. The detailed description of the most critical parts of the Matlab code’s implementation is provided in Section 4, followed by test examples in Section 4 where the ease of use of the code is presented.

## 2. Optimal Design of Materials

**Figure 1:**
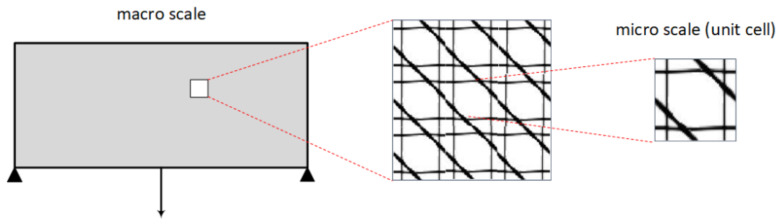
Schematic representation of the periodic unit cell (micro scale) inside the macro structure (macro scale).

The formulations of the topology optimization (TO) problem used for the design of materials are expressed as the optimal distribution of material volume fraction into the unit cell design domain so that the structural response is optimized. Thus, compared to the original TO problem, the design variables are different, from density values *X* of the finite elements used to discretize the macro design domain to densities *x* of the finite elements discretizing the micro-unit cell design domain. In this scope, it can be seen that the optimization procedure performed involves two different scales; the macro scale and the micro one (Figure 1). The design variables as well as the volume constraint are defined at the micro scale, where as the objective function is set on the macro scale; however, it is still expressed as a function of *x*. The transition between the two scales is achieved through the elasticity tensor by means of the homogenization method [38].

The mathematical formulation of the typical topology optimization problem is thus changed according to the following expression of Equation (1).

(1) $$\begin{array}{cc} C(x) & =F^{T}\cdot U\left(x\right)\\ s.t.\\ F & =K\cdot U(x)\\ V\left(x\right)/V_{0} & =f\\ 0\leq & x_{e}\leq 1 \end{array}$$

where C(x) is the compliance, *F* is the load vector, U(x) is the resulting displacements from the structural analysis, F=K·U is the linear system of equations derived from the finite element method, V(x) is the material volume resulting from the densities *x*, $V_{0}$ is the full domain material volume and *f* is the volume fraction applied as a constraint. The derivative of the objective function is obtained using the adjoint method [41] as described in the following expression of Equation (2):

(2) $$\frac{\partial C}{\partial x_{e}}=-U^{T}\cdot \frac{\partial K}{\partial x_{e}}\cdot U$$

while $\partial K/\partial x_{e}$ is calculated using the following expression of Equation (3):

(3) $$\frac{\partial K}{\partial x_{e}}=\frac{\partial C^{H}}{\partial x_{e}}\cdot K_{0}$$

where $C^{H}$ is the homogenized elasticity tensor. According to the homogenization theory, the elasticity tensor is obtained by applying unit strains globally to the unit cell domain as well as locally to the finite elements used to discretize the unit cell domain and then using the following expression of Equation (4).

(4) $$C_{i,j}^{H}=\frac{1}{V}\sum_{e=1}^{N}\int_{x_{e}}\left(u_{e}^{0(i)}-u_{e}^{\left(i\right)}\right)\cdot k_{e}^{0}\cdot \left(u_{e}^{0(j)}-u_{e}^{\left(J\right)}\right)dV_{e}$$

where superscript 0 denotes the globally applied strains, thus $u^{0}$ are the displacement fields resulting from the globally applied unit strains, *u* are the displacement fields resulting from the locally applied unit strains and $C^{H}$ is the elasticity tensor. If Equation (4) is then differentiated with respect to $y_{e}$, the derivative of the elasticity tensor can be obtained using the following expression of Equation (5):

(5) $$\frac{\partial C^{H}}{\partial x_{e}}=\frac{\partial E}{\partial x_{e}}\cdot \frac{1}{V}\int_{x_{e}}\left(u_{e}^{0(i)}-u_{e}^{\left(i\right)}\right)\cdot k_{e}^{0}\cdot \left(u_{e}^{0(j)}-u_{e}^{\left(J\right)}\right)dV_{e}$$

Furthermore, the derivative of the Young modulus with respect to each unit cell element density is obtained using the modified SIMP approach [42]. Thus, the Young modulus as a function of density value *x* is defined using the following expression of Equation (6):

(6) $$E\left(x\right)=E_{min}+x^{p}\cdot \left(E_{0}-E_{min}\right)$$

and the derivative from the expression of Equation (7):

(7) $$\frac{\partial E}{\partial x_{e}}=p\cdot x^{p-1}\cdot \left(E_{0}-E_{min}\right)$$

## 3. Model Order Reduction in Material Optimization

The main focus of the model order reduction (MOR) approaches is to reduce the computational cost required for solving the linear system of equations formulated from the finite element method (FEM). This is achieved by creating a reduced basis model and finding an approximate solution instead of solving the full-order system of equations. The creation of the reduced basis system of equations is accomplished by substituting first the displacement vector *U* in the finite element equilibrium system of equations with an approximation vector Φ·y. Matrix $\Phi =\{\Phi_{i},\cdots ,\Phi_{m}\}$ consists of the reduced basis vectors, and its first dimension corresponds to the dimension of vector *U*. Its second dimension refers to a small number (e.g., 5 to 10) chosen as the number of the reduced basis vectors. The left and right hand sides of the resulting equation is then multiplied by $\Phi^{T}$, as shown in the following expressions of Equation (8).

(8) $$\begin{array}{c} F=K\cdot U⇌\\ F\approx K\cdot \Phi \cdot y⇌\\ \Phi^{T}\cdot F\approx \Phi^{T}\cdot K\cdot \Phi \cdot y⇌\\ F_{eff}\approx K_{eff}\cdot y \end{array}$$

where *F* is the load vector, *U* is the displacement vector, *K* is the stiffness matrix, $F_{eff}$ is the reduced approximation of the load vector, $K_{eff}$ is the reduced approximation of the stiffness matrix and *y* is the reduced basis displacement vector. In general, to assess the accuracy of the projected solution, the residual load can be obtained and divided by the norm of the original load vector, as shown in the following Equation (9).

(9) $$e^{2}=\frac{{∥K\cdot \Phi \cdot a-F∥}^{2}}{{∥F∥}^{2}}$$

Thus, the most important part of the procedure is the creation of the reduced basis vectors that represent the main variation in most of the reduced model approaches. In the following subsections, the methodology as well as the creation of the reduced basis vectors of three MOR approaches will be presented. The three approaches implemented are the proper orthogonal decomposition (POD), the on-the-fly reduced order model construction and the approximate reanalysis following the combined approximations approach. In most MOR approaches, the approximation of the displacement field is taken into account in the calculation of the sensitivities through the expression of Equation (10).

(10) $$\frac{\partial C}{\partial x_{e}}=-y^{T}\cdot \Phi^{T}\cdot \frac{\partial K}{\partial x_{e}}\cdot \Phi \cdot y-\sum_{i=1}^{N_{b}}\lambda_{i}^{T}\cdot \frac{\partial K_{i}}{\partial x_{e}}\cdot U_{i}$$

where the first term of the expression corresponds to the sensitivity calculated from the approximate solution and the second part denotes the adjustment term which corrects the sensitivity calculation, taking into account that the solution is a approximation. The term $\lambda_{i}$ is the solution vector of the following expression of Equation (11) for each reduced basis vector.

(11) $$K_{i}\cdot \lambda_{i}=2\cdot y_{i}\cdot \left(F-K\cdot \Phi \cdot y\right)$$

$U_{i}$ used in Equation (10) and $K_{i}$ of Equation (11) denote the displacement vector and stiffness matrix of each reduced basis vector, respectively. Aiming to simplify the code implementation of the adopted MOR approaches into the optimal material design procedure that is described below, the sensitivity adjustment term in not taken into account.

### 3.1. Proper Orthogonal Decomposition

According to the proper orthogonal decomposition (POD) approach, the construction of the reduced basis vectors is achieved by means of the singular value decomposition (SVD) factorization methodology. In particular, a small number (e.g., five to ten) of optimization iterations is performed first that is equal to the number of the reduced basis vectors, in which the full-scale system equations are solved and the resulting displacement vectors are stored in the matrix *A*. Thus, matrix *A* is composed of different snapshots of the displacement field in the early phases of the optimization procedure. Before applying the SVD factorization methodology to the matrix *A*, the mean of the displacement snapshot of the final reduced basis vector is subtracted from *A*, as described in [22]. Then, SVD methodology is applied to matrix *A* and three different matrices are generated, as shown in the following Equation (12):

(12) $$A=\bar{U}\cdot \Sigma \cdot V^{{}^{\prime }}$$

Matrix $\bar{U}$ in general contains information about the spacial correlation of the snapshots of matrix *A*. Matrix Σ is a diagonal matrix containing the weight coefficients denoting the importance of each column of matrix $\bar{U}$, and finally *V* contains the corresponding time dynamics of each of the columns of matrix $\bar{U}$. The columns of matrix $\bar{U}$ are also called the POD modes and are used as the reduced basis vectors $\Phi_{i}$ consisting of matrix Φ. Thus, in the POD approach, the reduced basis matrix Φ coincides with the first matrix (i.e., $\bar{U}$) of the SVD of matrix *A*.

(13) $$\Phi =\bar{U}$$

Subsequently, given the creation of the reduced basis matrix Φ in each optimization iteration, the displacement vector is obtained using the constructed reduced model and then projected to the full scale. The accuracy of each new solution in validated using Equation (9), and if the deviation is too large, a full-scale finite element analysis (FEA) is performed and the matrix *A* is updated with the new snapshot of displacements (mth column of matrix *A*), removing the earliest generated one (i.e., the first column of matrix *A*). A new SVD is performed on the updated variant of *A* and a new reduced basis matrix Φ is used for the next iterations.

### 3.2. On-the-Fly Reduced Order Model Construction

Similarly to the POD approach, according to the on-the-fly approach a number of optimization iterations are performed first in order to generate displacements snapshots of the early optimization stages. Then, the reduced basis vectors are created based on the Gram–Schmidt orthogonalization methodology which is applied onto the displacement snapshots of the early optimization stages, as follows: for the first reduced basis vector, only the first displacement snapshot is utilized, in which a normalization is performed following the expression of Equation (14):

(14) $$\Phi_{1}=\frac{U_{1}}{∥U_{1}∥}$$

For the next reduced basis vectors, the following Gram–Schmidt orthogonalization procedure is applied, taking into account all previous reduced basis vectors, as shown in the following expression of Equation (15).

(15) $${\hat{\Phi }}_{i+1}=U_{i+1}-\sum_{j=1}^{i}\langle U_{i+1},\Phi_{j}\rangle \Phi_{j}$$

Subsequently, the new ${\hat{\Phi }}_{i+1}$ is normalized (as denoted in Equation (16) and the resulting vector is added to the reduced basis matrix.

(16) $$\Phi_{i+1}=\frac{{\hat{\Phi }}_{i+1}}{∥{\hat{\Phi }}_{i+1}∥}$$

Following the same steps as described for the POD approach, when the reduced basis matrix Φ is constructed, the subsequent optimization iterations rely on approximate displacement fields obtained using the reduced basis matrix and the accuracy of every new reduced basis based FEA in assessed using the expression of Equation (9). If the accuracy is not acceptable, a new reduced basis vector is created by means of a full-scale FEA and using the previously described procedure. Then, matrix Φ is updated by removing the earliest generated reduced basis vector (i.e., first column of matrix Φ) and adding the new one as the mth column of matrix Φ.

### 3.3. Approximate Reanalysis

In contrast to the POD and on-the-fly approaches, in the approximate reanalysis, one the reduced basis vectors is not created based on displacement snapshots obtained from the initial optimization iterations. Instead, new reduced basis vectors are created in each optimization iteration. These reduced basis vectors are based only on a single snapshot of the displacement field obtained by solving the full-scale system of equations; the displacement field snapshot is updated during the optimization procedure. The fist reduced basis vector is equal to the displacement snapshot used as the basis of the reduced order model, and thus is defined using the following expression of Equation (17):

(17) $$\Phi_{1}=U_{1}=K_{0}^{-1}\cdot F$$

Using $\Phi_{1}$ and $K_{0}$ as the basis of each reduced basis matrix Φ, at each iteration, a new set of reduced basis vectors is constructed. Each vector is obtained using the following expression of Equation (18):

(18) $$K_{0}\cdot U_{i}=F-\Delta K\cdot U_{i-1}\;i=2\ldots i_{max}$$

where $\Phi_{i}=U_{i}$, ΔK is the difference between the original stiffness matrix $K_{0}$ and the one corresponding to the current iteration. The size of the reduced basis is not the same for every iteration; after the creation of each reduced basis vector the accuracy of the solution is validated using Equation (9), and if it is below a certain threshold, the accuracy of the solution is accepted. A maximum size of reduced basis vectors is also provided. The update of the first reduce basis vector is usually performed after either a fixed number of iterations or after the change in the design variables or the compliance is significant. For a more detail review of the approximate reanalysis approach, the reader is referred to [15].

## 4. The Matlab Code Implementation

Part of the implementation of the methodologies described previously into a Matlab code is based on two existing codes. For the homogenization part, the basis was the Matlab code presented by Andreassen in [38], whereas for the topology optimization part, the basis was the Matlab code presented also by Andreassen in [25]. For efficiency, in the following sections only the parts of the code modified and the logic behind these modifications will be presented, starting from the part of the homogenization method and then to the topology optimization part. The code implementation presented here is composed of nine Matlab files. These are: homogenize function (i.e., *homogenize.m* Matlab file) that implements the homogenization procedure, elementMatVec function (i.e., *elementMatVec.m* Matlab file) that is used to compute the element load vectors and stiffness matrix, Q4elementStiffnessMatrix function (i.e., *Q4elementStiffnessMatrix.m* Matlab file) that is used for performing a similar role to the elementMatVec function, interpolate function (i.e., *interpolate.m* Matlab file) that performs the SIMP interpolation scheme, UCOpt function (i.e., *UCOpt.m* Matlab file) that performs the material topology optimization procedure, and three additional Matlab files containing the procedures of the corresponding MOR approaches, i.e., *pod.m* Matlab file containing the pod function, *onthefly.m* Matlab file containing the onthefly function and *ar.m* Matlab file containing the ar function.

### 4.1. Homogenization Code Implementation (Matlab File “homogenize.m”)

In this section, the modifications made to the homogenization Matlab files will be presented. For a more in-depth description of the functionalities of the original Matlab homogenization code, the reader is referred to [38]. There were two main modifications of the current implementation compared to the original function, denoted as homogenize, that is used for implementing the homogenization method, originally presented by Andreassen [38]. The first modification refers to the transition from the lame parameters to the Poisson ratio and Young modulus parameters, and the second one to the addition of the derivative of the homogenized tensor $dC^{H}/dy_{e}$ with respect to the densities at the unit cell level.

Input parameters: The input arguments of the new implementation of the homogenize function are the following:

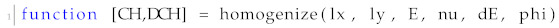



where lame parameters as well as the mapping parameters are replaced by matrix *E* containing the values of the Young modulus for every finite element used to discretize the unit cell domain, matrix dE contains the derivative of the Young modulus based on the modified SIMP approach, following the expression of Equation (7) and the Poisson ratio nu that is the same for all finite elements. In addition, an extra output argument was added to the method called DCH which is the derivative of the elasticity tensor from the expression of Equation (5), calculated using the parameter matrix dE.

Initialization: Due to the elimination of the mapping variable, the number of elements along the directions of the abscissa and ordinate of the unit cell are taken from the size of matrix *E*, and thus Line 4 (of the original code in [38] function) was slightly modified to take the number of elements from matrix E, as follows:

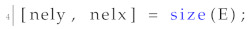



By using the Young modulus and Poisson ratio, the need for decomposing into two parts the loading vectors and stiffness matrix as described in [38] is not required. Thus, function elementMatVec was modified to return three loading vectors corresponding to the three different unit strains, as shown in the following expression of Equation (19) and the element stiffness matrix computed using the Poisson ratio parameters without the Young modulus.

(19) $$f_{e}^{i}=\int_{V_{e}}B_{e}^{T}\cdot C_{e}\cdot e^{i}dV_{e}$$

Thus, Line 9 (of the original code in [38]) was modified to have the following form:

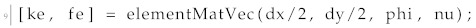



Assembly of the stiffness matrix and loading vectors: elementMatVec function (see Matlab file “*elementMatVec.m*”) was modified to compute the element loading vectors and stiffness matrix using the Poisson ratio by changing the first Line of the function to compute the elasticity tensor from Poisson ratio and Young modulus of one instead of the Lame parameters as presented below:

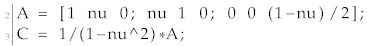



Thus, in the last lines of the function where the loading vectors and stiffness matrix are computed, the lines are

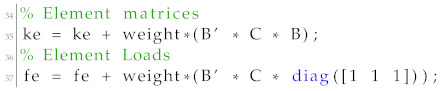



In the assembly of the global stiffness matrix part of the homogenize function, Lines 34 and 35 (of the original code in [38]) are removed due to Young modulus already being a matrix, and Line 37 (of the original code in [38]) is modified to multiply the element stiffness matrix with a vector of the Young modulus, as shown below:

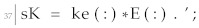



Moving now to the creation of the global loading vector, Line 41 (of the original code in [38]) is replaced by a simple multiplication of the element loading vector with the element Young modulus.

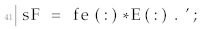



Due to the element loading vectors as well as the element stiffness matrix no longer being separated into two parts, Lines 53 and 54 (of the original code in [38]) are removed from the code. In addition, an extra line is added bellow the initialization of the elasticity tensor, initializing the derivative of the elasticity tensor. During the iterative procedure performed from Lines 64 to 75 (of the original code in [38]), the parameters sumLambda and sumMu are replaced with the parameter sumYoung which is obtained in the same way using ke instead of keLambda and keMu. An extra procedure is added to compute the derivative of the elasticity tensor in which the variable sumYoung is multiplied with the derivative of the Young modulus and then added to the cell variable DCH. DCH is a cell variable of a size of the number of elements, and contains the 3×3 derivative of the elasticity tensor of each element. The new iterative procedure is presented below:

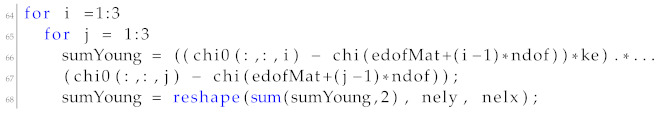

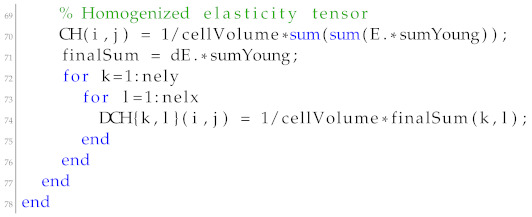





### 4.2. Topology Optimization Code Implementation

In this section, all modification applied to the top88 code published in [25] will be presented. The aim of all modifications was to transfer the code implementation from the conventional topology optimization formulation into the optimal design of materials. The new function used to perform the optimization procedure is called UCOpt. In this function, in addition to the input parameters already present in the original top88 code, four extra parameters were added. Due to the two different scales (micro and macro), two parameters (i.e., lx, ly) representing the dimensions along the directions of the abscissa and ordinate of the macro domain, respectively, were added for the case of macro scale, and two parameters (i.e., nlx, nly) representing the number of elements along the directions of the abscissa and ordinate of the unit cell were added for the micro scale. Thus, the resulting function is presented below:

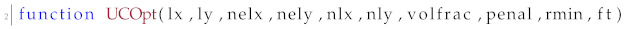



**Figure 2:**
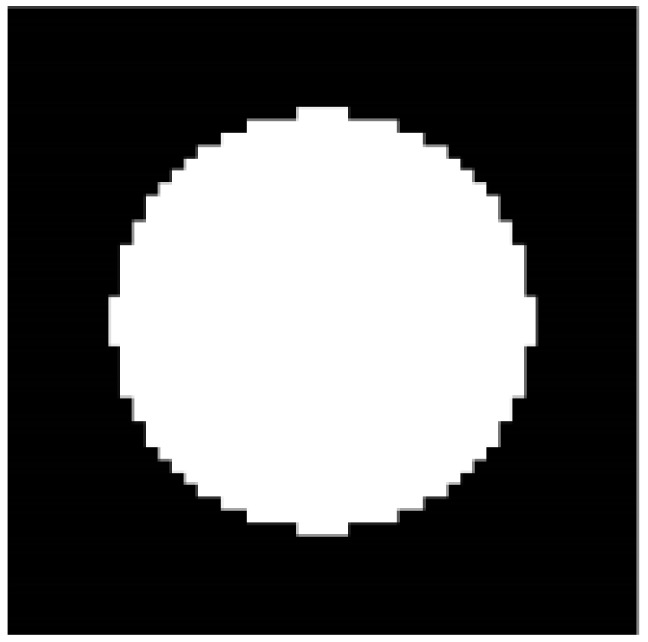
Initial unit cell.

As for the creation of the stiffness matrix variable KE, a new function is utilized. This function takes into consideration the length of the finite element along the directions of the abscissa and ordinate in the form of dx and dy, as well as the elasticity tensor instead of the Young modulus and the Poisson ratio used in the original code. This change is applied to enable the creation of the stiffness matrix from the homogenized elasticity tensor created by the homogenization function. In addition, the creation of the element stiffness matrix is performed inside the optimization procedure before the finite element analysis. Moving to the initialization of the design variables, in Lines 40 to 47 (of the UCOpt function), the initialization of the design variable is performed, in which instead of mapping the volume fraction to all densities and circle of zero densities is created in the centre of the unit cell, and all other densities are set to one, as shown in Figure 2.

This is achieved using the following iterative procedure:

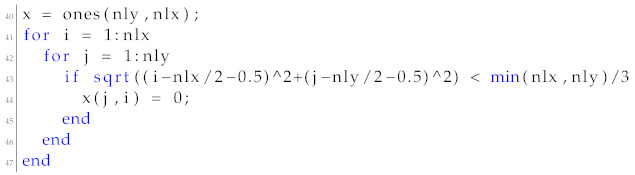



Moving to the optimization loop, two additional steps are added before performing the finite element analysis part. In the first step, a function called interpolate is utilized to compute the Young modulus and its derivative with respect to the design variables using the expressions of Equation (6) for the element Young modulus and Equation (7) for the corresponding derivative. During the second step, the resulting Young modulus and its derivative are provided to the homogenize function, which in turn produces the elasticity tensor and its derivative for each element consisting the unit cell. Moving to the finite element analysis part, the function Q4elementstiffnessMatrix is utilized to obtain the element stiffness matrix from the homogenized elasticity tensor, which in turn is used to perform the finite element analysis and compute the macro-domain displacements. For the computation of the sensitivities, an iterative procedure is utilized, looping for each element of the micro domain to create a different stiffness matrix for each unit cell element based on each element’s derivative of the homogenized elasticity tensor. Then, the variable ce is computed in the same manner as in the original code, resulting in the computation of the derivative dc.

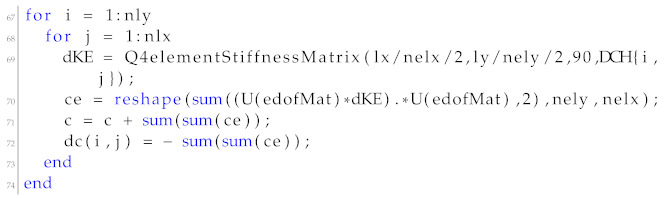



### 4.3. Model Order Reduction: Code Implementation

In this section, the implementation of the three model order reduction approaches will be presented. Aiming to create an easy integration of the three approaches into the UCOpt function presented in the previous section, the usage of the class structure is opted for the three MOR approaches. Thus, each approach is created as a single Matlab class object containing three common functions denoted as solve, fea and counts, respectively. The solve function is implemented differently for each class, while it is used by the UCOpt function in order to compute every set of displacements during the optimization procedure. The two other functions are the same for all three classes and they are used to perform the full-scale finite element analyses (function fea) and to return the number of full- and reduced-scale iterations performed (function counts). Since the class properties are modified during the optimization procedure, all MOR classes inherit from the handle a Matlab class. In order to use the MOR classes in the UCOpt function, an extra parameter is used called *p*, representing the MOR class, while Line 62 (of the pod class) is modified to call the solve function of the *p* class, as presented below:

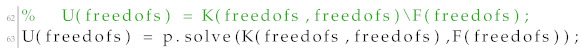



#### 4.3.1. POD: Code Implementation (Matlab File “pod.m”)

The pod class was developed for the code implementation of POD approach, which except for the constructor function, requires seven properties and three functions. Out of these properties, three refer to iteration trackers, i.e., parameters labeled as loop, fll and rdc tracking the total number of TO iterations performed, the total number of full finite element analyses and the total number of reduced basis finite element analyses, respectively. The forth parameter refers to the tolerance tol that represents the residual force tolerance used as a criterion for updating the reduced basis vectors after the creation of the reduced basis. The remaining three parameters correspond to the number of the reduced basis vectors Nb, the reduced basis matrix fi and a matrix containing the displacement snapshots *A* that is used to create the reduced basis matrix.

The implementation of the POD approach is performed inside the solve function. The solve function is separated into two main sections. The first section is executed during the first iterations for creating the first variant of the reduced basis matrix fi, as shown below:

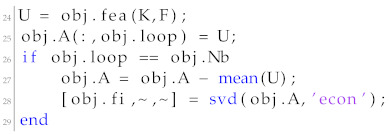



The second section is executed after the first creation of the reduced basis. In particular, in this section the reduced displacement field *y* is calculated, projecting it to the full scale of the displacement field *U*. Then, it is determined if the forces residual is acceptable. If the forces’ residual is deemed not acceptable, then the reduced basis is updated using a new set of displacement snapshots. The implementation is presented below:

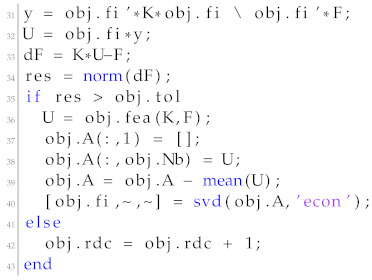



For the creation of the reduced basis matrix fi, the svd function of Matlab is utilized selecting the ${}^{\prime }econ^{\prime }$ option for generating an economy-size decomposition of matrix *A*.

#### 4.3.2. On-the-Fly: Code Implementation (Matlab File “onthefly.m”)

In the implementation of the on-the-fly reduced order model approach, the number of properties required by the corresponding class is reduced from seven to six, basically removing only the displacement snapshot matrix *A*. All other properties remain the same as those used in the POD implementation of the corresponding class. In the same manner as in the POD class, the implementation of the on-the-fly approach requires the use of the solve function. The on-the-fly implementation is also separated into two main parts. In the first one where the reduced basis matrix fi is computed, the norm function of Matlab is utilized to perform the normalization of the displacement field vector, whereas the procedure is exactly as described in Section 3.2 where the theoretical description of the on-the-fly reduced order model approach is provided.

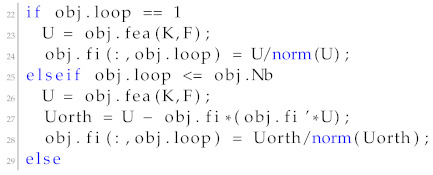



In the second part of the on-the-fly implementation, the procedure mirrors that of the POD implementation where instead of the svd function of Matlab, the update of the reduced basis matrix is performed as described in the expressions of Equations (15) and (16) in Lines 42 to 45 (of the onthefly class).

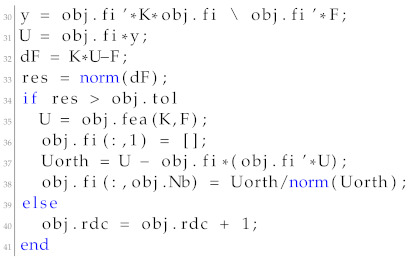



#### 4.3.3. Approximate Reanalysis: Code Implementation (Matlab File “ar.m”)

To keep the same structure of the code implementation for the approximate reanalysis approach as that of the previously presented two MOR approaches, the displacement snapshots are updated in a fixed number of iterations without taking into account the change in the objective function or the design variables. For the implementation of the approximate reanalysis approach, two new properties were added compared with the implementation of the on-the-fly approach. These properties correspond to the stiffness matrix K0 of the full-scale FEA and to a counter rf that keeps record of how often a new full-scale FEA will be performed.

As far as the solve function goes, its first part, i.e., Lines 32 to 35 (of the ar class), deals with the initialization of the reduced basis matrix fi. As discussed earlier, at the beginning of this section the criterion for the update of this procedure is simplified. More specifically, the update of the reduced basis matrix takes place in the first iteration and then after a fix number of iterations specified by the class variable rf, as shown bellow:

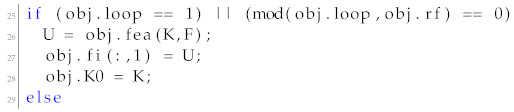



In the second part of the solve function, the reduced displacement vector is obtained. In more detail, in Line 37, the difference between the stiffness matrices (dK) is computed. Then, a while loop is implemented (see Lines 41 to 49 of the ar class) which builds the reduced basis matrix fi until either the maximum number of reduced basis vectors is reached or the residual is smaller than the tolerance value tol predefined.

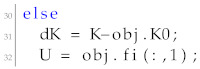

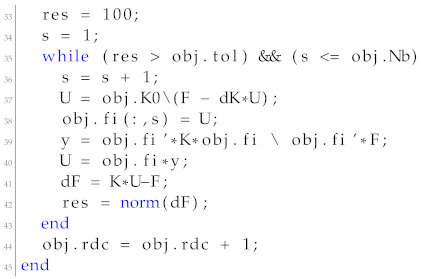





## 5. Test Examples

**Figure 3:**
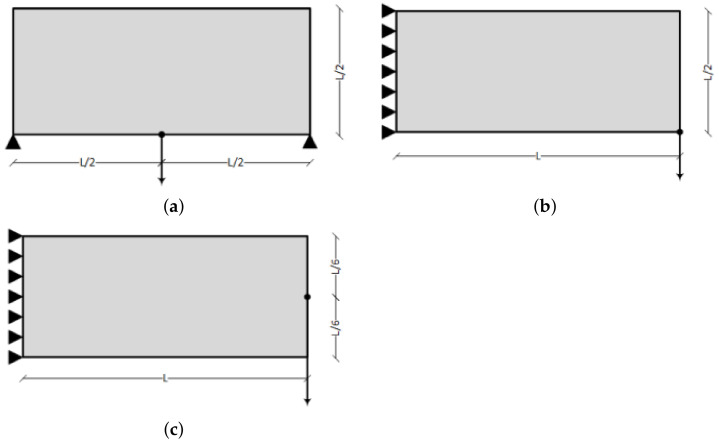
Test examples considered. (**a**) Bridge test example. (**b**) Cantilever beam 1 test example. (**c**) Cantilever beam 2 test example.

In this section, three simple test examples will be presented in order to demonstrate the ease of use of the proposed topology optimization Matlab code and how the three MOR approaches are integrated in order to assist the search procedure. The first test example refers to a simple bridge problem, the second one refers to the cantilever beam problem and the third one corresponds also to a cantilever beam problem with the load applied at the central right side of the domain. The macro domains for all test examples are schematically presented in Figure 3. In all test examples, the number of the iterations required, the number of full-scale FEAs and the final objective function value achieved are presented when the three MOR approaches are implemented, as well as the case without the application of any MOR approach.

### 5.1. Bridge Test Example

The implementation of the MBB beam test example with respect to the load vector and fixed degrees of freedom is the default implementation of the UCOpt function. The optimization parameters were a grid of 300×150 finite elements in the directions of the abscissa and ordinate for the discretization of the macro domain and a grid of 50×50 finite elements in the directions of the abscissa and ordinate for the discretization of the micro domain. A target volume fraction of 40%, penalization factor for the SIMP approach of 3, filter radius of 1.5 and application only of a sensitivity filter (option ft=1) were chosen. As far as the three MOR approaches go, the size of the reduced basis was chosen to be 8 for the POD and on-the-fly approaches and 10 for the approximate reanalysis, the tolerance for the update was set equal to 0.01 for all approaches and the update frequency for the approximate reanalysis was set to every six iterations. The script implementation for the POD-assisted optimization implementation is presented below:

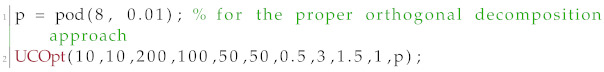



For the implementation of the other two MOR approaches, changes are only required in the first line where the MOR is created, as follows:

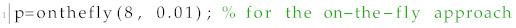



and

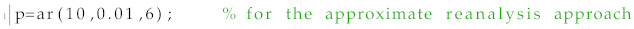



The results obtained of every MOR approach as well as the implementation without MOR assistance are presented in Table 1.

**Figure 4:**
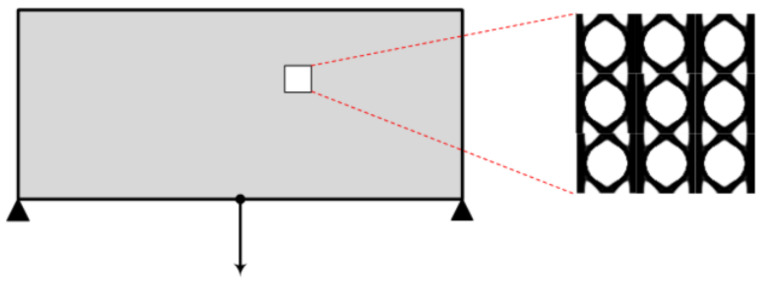
Optimized periodic unit cell for the bridge test example macro structure.

On the results of topology optimization achieved, in terms of unit cell structure, for the various implementations (with and without MOR), minor differences are observed, while the compliance value resulting from the four implementations is the same. Thus, only one of the results achieved, and in particular, the one obtained by means of the POD approach, is presented in Figure 4.

### 5.2. Cantilever Beam 1 Test Example

For the implementation of the first cantilever beam test example, changes to the load vector and fixed degrees of freedom should be made. In more detail, Lines 13 and 14 of the UCOpt function should be changed, as presented below:

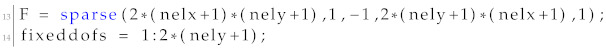



The optimization parameters were a grid of 200×100 finite elements in the directions of the abscissa and ordinate for the discretization of the macro domain and a grid of 50×50 finite elements in the the directions of the abscissa and ordinate for the discretization of the micro domain. Similarly to the first test example, a target volume fraction of 50%, penalization factor for the SIMP approach of 3, filter radius of 1.5 and application only of the sensitivity filter (i.e., option ft=1) were chosen. For the MOR approaches, the size of the reduced basis was chosen to be 4 for the POD and on-the-fly approaches and 10 for the approximate reanalysis, the tolerance for the update was set equal to 0.01 for all approaches and the update frequency for the approximate reanalysis was set to every five iterations. The results obtained for the first cantilever beam test example are presented below in Table 2.

**Figure 5:**
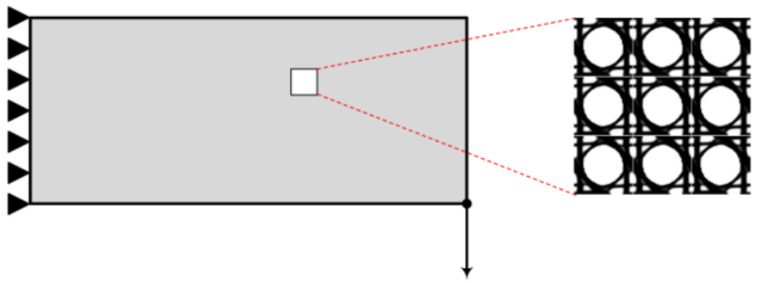
Optimized periodic unit cell for the cantilever beam 1 test example macro structure.

Similarly to the first test example, on the results of topology optimization achieved, in terms of unit cell structure, for the various implementations (with and without MOR) minor differences are observed, while the compliance value resulting from the four implementations is the same. Thus, only one of the results achieved, and in particular, the one obtained by means of the on-the-fly approach, is presented in Figure 5.

### 5.3. Cantilever Beam 2 Test Example

For the implementation of the second cantilever beam test example (labeled as cantilever beam 2), changes to the load vector and fixed degrees of freedom should be applied. In more detail, Lines 13 and 14 (of the UCOpt) function should be changed as presented below:

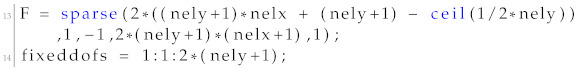



The optimization parameters were a grid of 300×100 finite elements in the directions of the abscissa and ordinate for the discretization of the macro domain and a grid of 50×50 finite elements in the the directions of the abscissa and ordinate for the discretization of the micro domain. Similarly to the first test example, a target volume fraction of 50%, penalization factor for the SIMP approach of 3, filter radius of 1.5 and application only of the sensitivity filter (i.e., option ft=1) were chosen. For the MOR approaches, the size of the reduced basis was chosen to be 4 for the POD and on-the-fly approaches and 10 for the approximate reanalysis, the tolerance for the update was set equal to 0.01 for all approaches and the update frequency for the approximate reanalysis was set to every five iterations. The results obtained for the second cantilever beam test example are presented below in Table 3.

**Figure 6:**
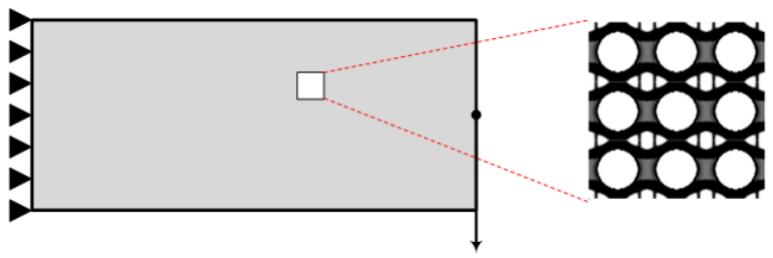
Optimized periodic unit cell for the cantilever beam 2 test example macro structure.

Similarly to the remarks reported for the first two examples presented before, on the results of topology optimization achieved, in terms of unit cell structure, for the various implementations (with and without MOR) observed minor differences are observed, while the compliance value resulting from the four implementations is the same. Thus, only one of the results achieved, and in particular, the one obtained by means of the approximate reanalysis approach, is presented in Figure 6.

## 6. Conclusions

The scope of this work is to present an open source numerical implementation of a methodology dealing with the optimal design of material structure using the theories of topology optimization and homogenization as well as the application of reduced order models. The code presented is written in Matlab, and two of the functions are partially based on existing well-known codes, published on the topology optimization and homogenization formulations. The implementation of the three model order reduction (MOR) approaches is simple, and the aim is to provide the means to integrate such models in any type of topology optimization problem formulation. Although the code implementation of the topology optimization part is based on a 2D space variant, it can easily be extended to the 3D space as well without the need to modify any of the three MOR classes presented in this study. The authors would be happy to receive suggested improvements that can be implemented in the public domain of the UCOpt codes.

## Figures and Tables

**Table 1:** Bridge test example: Results of each MOR approach as well as the classic implementation.

Approach	Total itrns	Full FEAs	Compliance
FEA	26	26	105.14
POD	26	8	105.14
on-the-fly	26	8	105.14
AR	26	5	105.14

**Table 2:** Cantilever Beam 1: Results of each MOR approach as well as the classic implementation.

Approach	Total TOP itrns	Full FEAs	Compliance
FEA	107	107	257.3
POD	104	20	257.3
on-the-fly	101	21	257.3
AR	107	22	257.3

**Table 3:** Cantilever Beam 2: Results of each MOR approach as well as the classic implementation.

Approach	Total TOP itrns	Full FEAs	Compliance
FEA	84	84	1369.0
POD	83	16	1369.1
on-the-fly	84	16	1368.9
AR	84	17	1368.9

## Data Availability

Please address the corresponding author by his e-mail address nlagaros@central.ntua.gr. The complete code, containing all participating functions (including the changes made to the original ones), is listed in https://github.com/nikoslagaros/TOPcodes, (accessed on 14 June 2022).

## Abbreviations

The following abbreviations are used in this manuscript:

BESO	Bi-directional Evolutionary Structural Optimization
FE	Finite Element
FEA	Finite Element Analysis
FEM	Finite Element Method
MOR	Model Order Reduction
POD	Proper Orthogonal Decomposition
SIMP	Solid Isotropic Material with Penalization
STO	Structural Topology Optimization
SVD	Singular Value Decomposition
TO	Topology Optimization
TOP	Topology Optimization Problem
